# Remarks on the History and Nature of the Dolor Faciei

**Published:** 1801-08

**Authors:** 

**Affiliations:** Wurzburg


### *Remarks on the History and Nature of the Dolor Faciei; by the late* Prof. SIEBOLD, of Wurzburg.*

THIS dreadful difeafe, which is commonly ftyled the *Dolor Faciei* of FOTHERGILL, on account of its being fuppofed to have been firft mentioned by that great practitioner, was before known, in the year 1756, to Mr. ANDREE, furgeon at Paris, who accurately defcribed it under the name of *tic*, and explained it with feveral obfervations.† Mr. SIEBOLD, however, traced the hiftory of this diforder farther back, finding, that in the year 1724, it had been treated by JOHN HARTMANN DEGENER, practitioner at Nimmegen, whofe excellent obfervation and defcription of that affection is communicated in the firft volume of the *Acta Naturæ Curiofor.* (*de dolore quodam perraro acerboque maxillæ finiftræ partes occupante et per paroxyfmos recurrente*, p. 347.) This practitioner even fuppofes, that LAWRENCE BAUSCH, phyfician at *Schweinfurth*, in Franconia, prefident and founder of the Societas Naturæ Curioforum, died of this diforder in the year 1665, according to the annals of that fociety.‡ The patient felt for four years a very excruciating pain in the right maxilla, fometimes growing lefs,

---

* Doloris Faciei, morbi rarioris atque atrocis obfervationibus illuftrati, adumbratio Diatribe I. qua exercitationes clinicas in nofocomio juliæo habendas indicit G. Ch. Siebold, Dr. Med. Prof. Wirceburgi, 1795, pp. 22 in 4to.—Diatribe II. qua pro capeffendo in illuftri inclyta et ornatiffima facultate medica loco et dignitate ad orationem die XXIX Jul. 1797, publice celebrandam invitat. pp. 23 in 4to.

† Obfervations Practiques fur les Maladies de l'urethre et fur les plufieurs faits convulfifs, à Paris 1756, pp. 318, &c.

‡ Mifcellanea Nat. Curios. Dec. I. Ann. II.

lefs, and then ceafing; but, at laft, it increafed to fuch a degree, that he became unable to fpeak or to fwallow, and notwithftanding all poffible remedies, died emaciated, and with a palfy of the left fide. There exift alfo, Obfervations on this difeafe by Dr. DANIEL LUDWIG, in the year 1673.*

The Dolor Faciei may be called analogically, (like otalgy, odontalgy, &c.) *profopalgy*, as it undoubtedly belongs to the topical pains without fever; and this name feems, befides, to be congruous with refpect to the feat as well as to the origin and kind of pain. The feat of the difeafe has been thought to originate in the teeth, but even drawing of them proved to be of no effect. It is obferved by Dr. FOTHERGILL, that the difeafe generally appears about the fortieth or fiftieth year of age, a period in which people are not much fubject to toothach; and though feveral patients fuffered tooth-ach at the fame time, yet others were quite exempt. The *antrum Highmori* is equally affigned as the feat of the difeafe, but without any probability, as it feems impoffible a pain fo fevere, and lafting for fo long a time, fhould take place here, without producing fome change, inflammation, and fuppuration, which never was the cafe in all the inftances of that painful affection, according to what we find recorded in the annals of medicine; teeth were drawn out, and an opening made into the *antrum*, without ever perceiving any matter to iffue thence. The pain generally extends itfelf to the jaws, a circumftance which is to be afcribed to the diftribution of nerves. The difeafe, luckily, occurs fo feldom, that even phyficians of the moft extenfive practice never had an opportunity of obferving it: We find it, however, mentioned by practitioners of all nations, except the Italians. Dr. FOTHERGILL met with fixteen inftances of it; Dr. THILENIUS, a German phyfician, faw it but twice during a moft extenfive practice of twenty years; Dr. AEPLI, a Swifs phyfician, only once in twenty-feven years. The following exhibits the literature of different nations on the above difeafe.

DUTCHMEN.—*Degener*, loc. citat.—*Van Wy* in Verhandelingen uitgegeven door het Zeeuwfch Genootfchap der Wetenschappen te Vliffingen, Dec. VII. 1782; i. e. Tranfactions of the Society at Vliffingen. Vol. VII.

FRENCHMEN.—*André*, loc. citat.—SAUVAGES; Nofologia Methodica, under the name of *Trismus Dolorificus*, Clafs IV. Ord. 1, Gen. 2, Spec. 14.—*Bonnard*; Journal de Medicine, 1778,

1778, month, July.—*Lavagan*; ibidem,—*Thouret*; Memoires de la Societé Royale de Medicine, à Paris, T. I, 1776, T. 3, 1779, T. 5, 1782 and 83. Journal Encyclopedique, m. April, 1777. Gazette Salutaire, No. 73. confer. RICHTER's Chirurgical Library, Vol. II, in German.—*Andry*; Mem. de la Soc. Roy. de Medicine, T. I. 1776, T. V, 1782 and 83. *Louis*; Gazette Salutaire, A. 1776, No. 36.—*Guerin*; Maladies des Yeux.—*Pujot*; Treatise on that disease of the face, which is named the Tic Douloureux, translated from the French into German, by Dr. Schreger, 1788.—*Spielmann*; Gazette Salutaire, 1791.—*Petit* and *Laugier*; Journal de Medicine, in July 1793.—*Waton*; ibid. 1793, March, No. I.

ENGLISHMEN.—*Fothergill*; Observations on the use of Hemlock, in Medical Observ. and Inquiries, Vol. III. &c. or in *Fothergill's* Medical and Philosophical Works; ed. *J. Elliot*, London, 1781, p. 315; of a painful affection of the face, ibid. p. 427; and Medical Observations and Inquiries, Vol. V. —*Davidson*; in *Duncan's* Medical Commentaries, Vol. V. 1792.—*Blunt*; Med. Observ. and Inq. Vol. V.

SWIZZERS.—*Rahn*; Museum der Heilkunde; i. e. Museum of Medicine, Vol. I.—*Aepli*; ibid. p. 302.—*Sauter*; ibid. also *Tissot* and *Pohlen*.—*Richts*; in *Sabatier* Traité Compl. d'Anatomie, T. III. p. 452.

GERMANS.—*Lentin*; first in *Blumenbach's Medicinische Bibliotheck*; i. e. Medical Library, Vol. I. (a periodical work, that is now discontinued) in his *Contributions to Practical Medicine*, (in German) Vol. I. 1797, p. 382—398. Vol. II. 1798, p. 92, seq. *Hufeland's* Practical Journal, Vol. IX. No. I. *conf.* Medical and Physical Journal, Vol. III. p. 575. —*Selle*; in *Neue Beitrage*, &c. i. e. New Contributions to Natural and Medical Science, Vol. I. p. 27, &c.—*Vogler*; *Blumenbach's* Medical Library, Vol. II. p. 506.—*Thilenius*; *Medicinische und Chirurgische Bemerkungen*; i. e. Medical and Surgical Observations: Franckfort, 1789, p. 283.—*Bohmer*; in Blumenbach's Medic. Libr. Vol. III. p. 315—336.—*Baldinger*; in his Medical Journal, Vol. II. p. 7.—*Leidenfrost* and *Gunter*; in *J. G. Forstmann* Differtatio de Dolore Faciei Fothergilli; Duisburg, 1794, 4to. extracted in *Tode's* Medical Journal, (in German) No. III. Vol. I.—*Richter* in his Surgical Library, Vol. XI. p. 135. (in German.)

FOTHERGILL observed, that this disorder mostly attacks women, but DEGENER and others saw it in men. Mr. SIEBOLD met with three instances in the female sex, and one in a man: It seems, however, to occur more frequently in women than in men, and the proportion is about five to four. It is remarkable, that the subjects of the disease in England were mostly moſtly women, in the proportion of 1 to 14; and in Germany moſtly men, in the proportion of 21 to 13. On reviewing the caſes mentioned by different authors, we obſerve, that they were for the moſt part confined to the period of age between the thirtieth and eightieth year; and inſtances of the diſorder being met with at an earlier period of life have but rarely occurred. The German tranſlator of Dr. Fothergill's works relates a caſe of a young lady, nineteen years of age; and alſo *Dr. Leidenfroſt* a ſimilar inſtance, (*Diſſert. ſupra citat.*) Dr. Rahn, Prof. Weidmann, of Mentz, and Prof. Siebold, of Wurzbourg, are the only perſons who have obſerved this diſorder in pregnant women. The patient of Prof. Weidmann was attacked by the pain a month before her delivery: evacuants were fruitleſſly employed, but ſhe was cured by opium and bark. Dr. Siebold's caſe is very remarkable, on account of the pain ceaſing entirely during pregnancy, a circumſtance that ſeems to prove the great power which the body enjoys in this ſtate, of reſiſting ſeveral diſeaſes, a remark already made by Hippocrates, (*de internis affectionibus*, cap. 53, ed. chart. t. viii. p. 677) who tells us, that a dolor iſchiadicus diſappeared during the period of pregnancy, which, however, recurred twenty days after the delivery. Dr. Sauter ſaw this painful affection diſappear during a putrid fever, and Dr. Selle in an intermittent fever, in both which caſes it returned after the fevers were cured. It ſeems not improbable, but that this diſorder may be endemical, and even epidemical; at leaſt the diſeaſe has not been more frequently obſerved than at *Clauſthal*, the capital of the Hercynian mountains; and in the mountainous country of *Salzbourg* it is not at all rare. Mr. Andre remarks, that in a very ſhort time he met with eight or ten caſes of this diſorder, whereas he had not a ſingle one afterwards during a period of twenty-ſeven years.

This affection ſeems not to be peculiar to a particular temper and habit of body, but attacks people of very different conſtitutions ſuddenly and unexpectedly: Its duration is extremely long, as it may laſt for many years without doing any evident harm to the whole conſtitution, or without terminating fatally in an immediate manner. A woman ſuffered this painful affection from her nineteenth year to her eightieth, and got rid of it but ſhortly before ſhe died. In other caſes, however, the diſorder emaciates and deſtroys the body, particularly for want of reſt: The diſeaſed ſide of the face is frequently disfigured, and perſons ſubject to this affection receive, as it were, a double profile, of which Prof. Baldinger relates a curious inſtance. (See his *Medical Journal*, Vol. II. No. 7.)

 Although

Although many fymptoms of this diforder are of the fpafmodic kind, yet we have more reafon to think it to belong to the clafs of convulfive diforders, and it is therefore erroneoufly called *trifmus dolorificus*, after SAUVAGE and PUGOL, becaufe the vehement contortions do not occur in every patient, and may, moreover, be rather confidered as *conamina naturæ medicatricis*, and efforts by which the patients endeavour to relieve themfelves from the pain, for which purpofe they likewife rub the part with much vehemence. A difficulty of fpeech and of fwallowing are obferved in fome cafes, probably from the diftortions of the mufcular fibres that are fubfervient to this purpofe. A confiderable falivation came on in another inftance, where the patient became almoft hectic; but Mr. SIEBOLD attended a patient whofe falival excretion was entirely furpreffed; another fubject of that diforder had a very much fwollen *glandula Warthoni*, from which a fœtid and purulent matter iffued. The eyes remain fometimes dry, and it is not always that the pain forces out tears; fometimes they become red and humid. In fome patients a rednefs and heat of the affected part precedes the paroxyfm of pain, of which phenomenon a curious inftance is related by Dr. RAHN, where the heat was fo intenfe, that it could be felt at fome diftance. Other fymptoms frequently to be remarked in patients of this kind are, an intumefcence of the belly, a torpor of the inteftinal canal, and an obftinate coftivenefs, which are probably owing to a fpafm of the *nervous fympatheticus magnus*, that is intimately connected by anaftomofis with the fifth pair, an opinion which agrees alfo fomewhat with analogy, as we do not rarely obferve, that if the cheeks, or the cheek-bones, are any way affected, the liver is alfo difeafed: Thofe fymptoms ought, therefore, to be rather confidered as merely fecondary, and arifing *per consensum*, than as the caufa primaria of the difeafe. It has been obferved in one cafe by Dr. LENTIN, that when the belly began to fwell, the pain foon after difappeared; and in another cafe it was greatly diminifhed by it, which fymptom feems to prove, that the morbid matter is depofited on the inteftines: it deferves, however, to be afcertained by farther obfervations. It is remarkable, that in a cafe of Dr. SIEBOLD, the patient had a particular relifh for fweet things, which that gentleman is inclined to afcribe to a depravation of tafte, occafioned by the affection of the neighbouring nerves, or to an obftruction of the liver, in which this fymptom fometimes occurs. The pulfe was often full, flow, and foft, during the paroxyfm, and at other times it decreafed ten beats at every return of the pain.

The feat of the pain has been obferved,

1. In

1. *In some single part of the face*, viz. at the inner *canthus* of the eye, FOTHERGILL; in the *orbita*, *the same*; at the *supercilia* of the right eye, PUGOL; in the jaw-bone, FOTHERGILL; in the joint of the jaws, SELLE, PUGOL; in the *maxilla inferior*, at the paſſage of the *nervus inframaxillaris*, LENTIN; in the *ossa temporum*, FOTHERGILL; in the *nervus infraorbitalis*, *Albinus* and *Van Wy*; in the *ala nasi*, VOGLER, THOURET; at the margin of the tongue, LENTIN.

2. *In one half of the face*, FOTHERGILL.

3. *In both cheeks*, PUGOL.

4. *In both sides of the maxilla inferior, at the exit of the nerve.*

5. *In the whole head and face*, LENTIN.

6. *In one foot*, LENTIN. (See Medical and Phyſical Journal, vol. iii. 575.)

The head and face are particularly expoſed to this painful affection, on account of the quantity of nervous fibres that are every where ſpread over the ſurface of the head. The pain eſſentially differs from any other that occurs in the various parts of the human body, though it may, in ſome meaſure, be compared with the *dolor ischiadicus* of COTUNNI. Dr. LENTIN derives its origin from the *medulla oblongata*; and Prof. SIEBOLD likewiſe thinks it may originate in a part remote from the affected place, in the *ganglion Gasseri*; others have derived it from the bones; but we ſtill want anatomical obſervations for aſcertaining the proper ſource of the pain.

*Remote causes*, *causæ prædisponentes*, of the diſorder appear to be, 1. Violent blows and contuſions of the above parts, ANDRE. 2. Cicatrices, LENTIN. 3. Preceding tooth-ach. 4. Too great tenderneſs of the ſkin; but the diſorder occurred in perſons, whoſe ſkin was by no means to be called tender or ſoft. 5. Congeſtions towards the head.

*Internal causes.* 1. Cancerous acrimony. Dr. FOTHERGILL firſt propoſed this opinion, whence it has been called by ſome authors, the *cancerous rheumatism.* The cancerous nature of the diſeaſe ſeems to be confirmed by the following arguments. 1. There is no other cauſe to be diſcovered, but that it is owing to a cancerous poiſon. 2. It moſt frequently occurs in women, who are particularly diſpoſed to cancers. 3. It generally appeared at the period of the ceaſing menſtruation, or when they were paſt the time of menſtruation, where a particular tendency to cancers take place. 4. The kind is extremely ſimilar to that occaſioned by a cancer. 5. Both diſorders have much analogy in their preceding ſymptoms. 6. Four women ſuffering this affection had the *scirrhus mammarum* previouſly. 7. The effect of hemlock in this diſeaſe, which is likewiſe much praiſed in cancers.

*Adverse arguments.* 1. The disease is not at all uncommon in men, where cancerous acrimony could not be traced as well as in many women. 2. Menstruation did not always cease in some patients, but they became even pregnant during the disease. 3. The cancerous poison easily attacks glandular parts; but we seldom find them altered, indurated, or painful in the neighbourhood of the pain. 4. There is no instance of the Dolor Faciei having terminated in a cancer of the lips or of the cheek. If it did however arise from a cancerous acrimony, it should be more frequently observed in men than women, because the *cancer labiorum* is oftener observed in the male sex. 5. The parts disposed to cancers have been frequently irritated by the persons who had the Dolor Faciei, without ever producing such an affection. 6. It is not probable that the cancerous acrimony should remain so long at a place so sensible, without manifesting itself in the general way.

These arguments are indeed of some weight, to make us believe that a cancerous poison is by no means the only cause of that affection, nay, that it is not even to be considered as a chief cause of it.

2. *Arthritic matter.* Almost all the authors who have treated on this subject, agree in assigning this as the proximate cause of that disorder, but particularly Dr. Leidenfrost. Dr. Degener is likewise of this opinion, having observed at the affected place a tumour similar to that in the joints of gouty people. Dr. Siebold saw in a patient suffering the most violent pains at the *supercilia*, a node perfectly like that of arthritic limbs; and Dr. Ludwig fairly tells us, that the patients suffering this disorder became gouty soon after. The disease seems to have an arthritic origin in the cases related by Dr. Boehmer and Lavagan, who saw the pain disappear when the usual arthritic paroxysms returned.

[ To be concluded in our next. ]

